# The impact of increasing levels of blood C-reactive protein on the inflammatory loci SPI1 and CD33 in Alzheimer’s disease

**DOI:** 10.1038/s41398-022-02281-6

**Published:** 2022-12-22

**Authors:** Jinghan Huang, Qiushan Tao, Ting Fang Alvin Ang, John Farrell, Congcong Zhu, Yixuan Wang, Thor D. Stein, Kathryn L. Lunetta, Joseph Massaro, Jesse Mez, Rhoda Au, Lindsay A. Farrer, Wei Qiao Qiu, Xiaoling Zhang, For the Alzheimer’s Disease Neuroimaging Initiative

**Affiliations:** 1 Departments of Medicine (Biomedical Genetics), Boston University School of Medicine, Boston, MA USA; 2 Departments of Pharmacology & Experimental Therapeutics, Boston University School of Medicine, Boston, MA USA; 3 Departments of Anatomy & Neurobiology, Boston University School of Medicine, Boston, MA USA; 4 Department of Epidemiology, Boston University School of Public Health, Boston, MA USA; 5 Department of Pathology and Laboratory Medicine, Boston University School of Medicine, Boston, MA USA; 6 Alzheimer’s Disease Research Center, Boston University School of Medicine, Boston, MA USA; 7 VA Boston Healthcare System, Boston, MA USA; 8 VA Bedford Healthcare System, Bedford, MA USA; 9 Department of Biostatistics, Boston University School of Public Health, Boston, MA USA; 10 Framingham Heart Study, Boston University School of Medicine, Framingham, MA USA; 11 Departments of Neurology, Boston University School of Medicine, Boston, MA USA; 12 Departments of Ophthalmology, Boston University School of Medicine, Boston, MA USA; 13 Departments of Psychiatry, Boston University School of Medicine, Boston, MA USA

**Keywords:** Genetics, Neuroscience, Personalized medicine

## Abstract

Apolipoprotein ε4 (*APOE* ε4) is the most significant genetic risk factor for late-onset Alzheimer’s disease (AD). Elevated blood C-reactive protein (CRP) further increases the risk of AD for people carrying the *APOE* ε4 allele. We hypothesized that CRP, as a key inflammatory element, could modulate the impact of other genetic variants on AD risk. We selected ten single nucleotide polymorphisms (SNPs) in reported AD risk loci encoding proteins related to inflammation. We then tested the interaction effects between these SNPs and blood CRP levels on AD incidence using the Cox proportional hazards model in UK Biobank (*n* = 279,176 white participants with 803 incident AD cases). The five top SNPs were tested for their interaction with different CRP cutoffs for AD incidence in the Framingham Heart Study (FHS) Generation 2 cohort (*n* = 3009, incident AD = 156). We found that for higher concentrations of serum CRP, the AD risk increased for SNP genotypes in 3 AD-associated genes (*SPI1*, *CD33*, and *CLU*). Using the Cox model in stratified genotype analysis, the hazard ratios (HRs) for the association between a higher CRP level (≥10 vs. <10 mg/L) and the risk of incident AD were 1.94 (95% CI: 1.33–2.84, *p* < 0.001) for the *SPI1* rs1057233-AA genotype, 1.75 (95% CI: 1.20–2.55, *p* = 0.004) for the *CD33* rs3865444-CC genotype, and 1.76 (95% CI: 1.25–2.48, *p* = 0.001) for the *CLU* rs9331896-C genotype. In contrast, these associations were not observed in the other genotypes of these genes. Finally, two SNPs were validated in 321 Alzheimer’s Disease Neuroimaging (ADNI) Mild Cognitive Impairment (MCI) patients. We observed that the *SPI1* and *CD33* genotype effects were enhanced by elevated CRP levels for the risk of MCI to AD conversion. Furthermore, the *SPI1* genotype was associated with CSF AD biomarkers, including t-Tau and p-Tau, in the ADNI cohort when the blood CRP level was increased (*p* < 0.01). Our findings suggest that elevated blood CRP, as a peripheral inflammatory biomarker, is an important moderator of the genetic effects of *SPI1* and *CD33* in addition to *APOE* ε4 on AD risk. Monitoring peripheral CRP levels may be helpful for precise intervention and prevention of AD for these genotype carriers.

## Introduction

Alzheimer’s disease (AD) is a neurodegenerative disorder with a long-term deteriorating process including memory decline, problems with language, disorientation, mood swings, loss of motivation, self-neglect, and behavioral issues [1]. It has been reported that over 5.8 million Americans aged 65 and older had AD dementia in 2020 [2]. Apolipoprotein ε4 (*APOE* ε4) is the largest genetic risk factor for late-onset Alzheimer’s disease (AD). A total of 40–70% of people with sporadic AD carry an *APOE* ε4 allele. However, not every person carrying AD risk variants develops AD. The AD risk potentially depends on both genetics and internal and external environmental components, such as proinflammatory factors, and their interactive effects on the disease [1].

Peripheral chronic inflammation has been linked to several age-related disorders, including cardiovascular disease and type 2 diabetes, both of which are associated with AD risk [3]. C-reactive protein (CRP) is an acute-phase protein secreted into blood and a biomarker for chronic low-grade inflammation. CRP levels increase in response to toxins or injuries in systemic inflammation and, more generally, with age [4]. Additionally, direct injection of CRP into the hippocampus of an AD mouse model enhanced the severity of AD-like pathology in the brain [5]. Previously, we observed that elevated blood CRP levels increased the risk of AD for people carrying the *APOE* ε4 allele [6–8]. In addition, low CSF CRP was the biomarker most closely associated with the *APOE* ε4 copy number, not high CSF CRP levels [9]. We hypothesized that CRP, as a key inflammatory element, could modulate the impact of other genetic variants on AD risk, especially variants in gene loci involved in proinflammation. Current genome-wide association studies (GWAS) have identified >50 AD loci, some of which are enriched explicitly in inflammatory pathways [10–12]. In this study, we selected ten common single nucleotide polymorphisms (SNPs) in reported AD risk loci encoding proteins related to inflammation/immune function, including clusterin (*CLU*, rs9331896) [13], Spi-1 proto-oncogene (*SPI1*, rs1057233) [14] and *CD33* molecule (*CD33*, rs3865444) [15–17]. We then tested the interaction effects between these SNPs and blood CRP levels on AD incidence as the primary outcome using the Cox proportional hazards model in two different cohorts following a meta-analysis of results from these two cohorts. The top findings were further tested in the ADNI cohort for the conversion of MCI to AD and AD-specific biomarkers measured from cerebral spinal fluid (CSF).

## Materials and methods

### Participants

#### UK Biobank (UKBB)

**Fig. 1:**
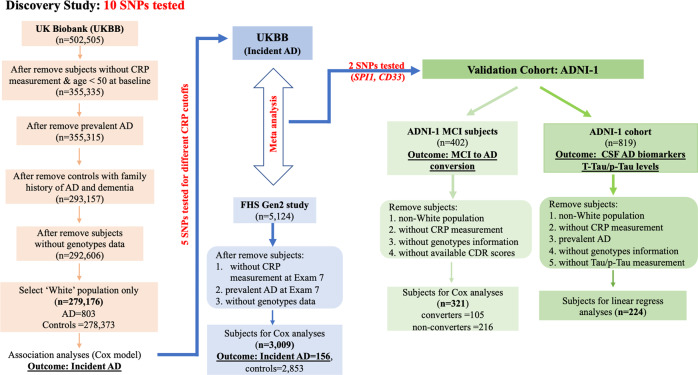
Study design and filters for UKBB, FHS and ADNI cohorts. The following three human datasets were analyzed in this study. *UKBB*: 279,176 participants (mean age 60.1 ± 5.5 years) of which baseline CRP was measured, and 803 incident AD cases were identified after 8.3 ± 0.9 years of follow-up. *FHS*: 3009 participants (mean age 60.8 ± 9.4 years) of which baseline CRP was measured (i.e., Exam 7), and 156 incident AD cases were identified after 14.9 ± 4.0 years of follow-up. *ADNI*: 321 MCI patients (mean age 75.0 ± 7.0 years) of which baseline CRP was measured, and 105 MCI-to-AD converters were identified after 31.8 ± 11.2 months of follow-up.

The UKBB contains genetic, biomarker, medical record and self-reported demographic and clinical information obtained from more than 500,000 persons living in the UK [18]. Included in this study were 279,176 self-reported white participants after excluding subjects who did not have baseline CRP measurement, lacked genetic information, had prevalent AD/dementia at baseline, were not at AD-risk age during the follow-up time due to a young age (i.e., age <50 years at baseline) or were nonwhite (self-reported) (Fig. 1). In addition, we excluded those controls who had a family history of AD or dementia because most UKBB participants did not undergo a formal neuropsychological examination and consensus diagnosis evaluation; thus, dementia in this group of subjects, who are considered in some studies as “proxy” AD cases [10, 19], is likely to include mixed diagnoses. Participants included in the analysis had an average age of 60.1 ± 5.5 years and were mostly female (53.9%) (self-reported), British based on genetic analysis of population substructure (94.1%), and cognitively normal (99.7% versus 0.3% diagnosed with AD with 8.3 ± 0.9 years follow-up on average). The AD status for each subject was determined by medical or hospital impatient record (i.e., ICD-10 diagnosis). The AD cases had ICD-10 codes of F00 (AD dementia) or G30 (AD). AD diagnosed cases were recorded until March 30, 2017, which was used as the end date for survival analysis of incident AD. The characteristics of the discovery sample are shown in Table 1.

**Table 1:** Basic characteristics, APOE, CRP measurement and incidence of AD in main discovery—UKBB.

Characteristic	All subjects	CRP ≥3 mg/L	*P* value^a^ (3 mg/L)	CRP ≥8 mg/L	*P* value^a^ (8 mg/L)	CRP ≥9 mg/L	CRP ≥10 mg/L	CRP ≥11 mg/L	CRP ≥12 mg/L
*N* subjects, No. (%)	279,176	67,273 (24.10)	NA	17,173 (6.15)	NA	14,519 (5.20)	12,335 (4.42)	10665 (3.82)	9177 (3.29)
Incidence of AD, No. (%)	803 (0.29)	196 (0.29)	0.87^b^	61 (0.36)	0.10^b^	50 (0.34)	47 (0.38)	40 (0.38)	37 (0.40)
Age when measuring CRP, mean (SD)	60.07 (5.47)	60.49 (5.43)	<0.001^c^	60.54 (5.47)	<0.001^c^	60.55 (5.46)	60.58 (5.44)	60.60 (5.45)	60.64 (5.44)
Age, range	50–73	50–72	NA	50–70	NA	50–70	50–70	50–70	50–70
AD onset age, mean (SD)	70.51 (4.49)	70.49 (4.06)	0.96^c^	71.20 (3.72)	0.14^c^	70.70 (3.57)	70.77 (3.64)	70.42 (3.66)	70.84 (3.29)
Follow-up years, mean (SD)	8.26 (0.92)	8.29 (0.92)	<0.001^c^	8.30 (0.93)	<0.001^c^	8.30 (0.93)	8.30 (0.93)	8.29 (0.93)	8.29 (0.93)
Female, No. (%)	150,542 (53.92)	39,363 (58.51)	<0.001^b^	10,171 (59.23)	<0.001^b^	8530 (58.75)	7178 (58.19)	6128 (57.46)	5226 (56.95)
Years of education, mean (SD)	18.13 (3.12)	17.56 (3.02)	<0.001^c^	17.44 (2.99)	<0.001^c^	17.47 (3.02)	17.47 (3.02)	17.48 (3.03)	17.49 (3.02)
*APOE* ε2^d^, No. (%)	37,330 (13.37)	9,889 (14.70)	<0.001^b^	2,656 (15.47)	<0.001^b^	2263 (15.59)	1928 (15.63)	1673 (15.69)	1452 (15.82)
*APOE* ε4^d^, No. (%)	67,590 (24.21)	12,487 (18.56)	<0.001^b^	2,970 (17.29)	<0.001^b^	2512 (17.30)	2118 (17.17)	1832 (17.18)	1583 (17.25)
CRP, median (IQR), mg/L	1.43 (0.72–2.90)	5.07 (3.77–8.11)	NA	12.47 (9.73–17.92)	NA	13.68 (10.84–19.45)	14.87 (11.95–21.08)	16.10 (12.96–22.47)	17.37 (14.09–24.11)

*AD* Alzheimer’s disease, *CRP* C-reactive protein, *APOE* apolipoprotein, *IQR* interquartile range.

A total of 279,176 subjects of UK Biobank (UKBB) were divided into different CRP cutoffs in the analysis. Mean ± SD were reported ANOVA test for continuous variables, while n (%) with χ^2^ test was used for binary variables. Raw P values for the comparisons between CRP < and ≥3 mg/L as well as < and ≥8 mg/L are shown.

^a^Raw *P* value of comparison between CRP groups < and ≥3 mg/L as well as < and ≥8 mg/L.

^b^χ^2^ test raw *P* value.

^c^T test raw *P* value.

^d^*APOE* ɛ2 = ɛ22 + ɛ23; *APOE* ε4 = ɛ34 + ɛ44.

#### Framingham heart study

The Framingham Heart Study (FHS) is a single-site, multigeneration, community-based, prospective cohort study of health in Framingham, Massachusetts. The current study focused on Offspring cohort (Generation 2) white participants who have data on GWAS and serum CRP measurement and have been rigorously evaluated for cognitive decline and dementia since 1979. Other details of this cohort have been previously described [20]. In brief, the cohort included 5124 white participants at the first health examination (1971–1975). The number of participants included in this study was 3009 (mean age 60.8 ± 9.4, 53.7% female [self-reported]) with CRP measured at examination 7, and 156 AD incident cases were collected through the period ending in 2016 (Fig. 1, Table S1). Participants were evaluated longitudinally for incident AD as previously described [8] using consensus diagnostic procedures [7].

#### Alzheimer’s disease neuroimaging initiative

ADNI-1 is a longitudinal multicenter study that was launched in 2003 as a public‒private partnership and designed to test whether serial magnetic resonance imaging (MRI), positron emission tomography (PET), other biological markers, and clinical and neuropsychological assessment can be combined to measure the progression of mild cognitive impairment (MCI) and early AD [21]. Participants underwent longitudinal in-depth neuropsychological evaluations [22], and consensus diagnoses of cognitive normal (CN), MCI, and AD were assigned based on established research diagnostic criteria [23]. Due to the small number of CN individuals with serum CRP measurements, participants with an MCI diagnosis at baseline were included with a follow-up of their MCI to AD conversion. After filtering out nonwhite subjects and those without CRP measurement, genotype information and CDR scores, 321 MCI patients were included in the analysis (Fig. 1). MCI to AD conversion was determined by comparing the baseline Clinical Dementia Rating (CDR = 0.5) with the most recent CDR score. MCI subjects whose most recent CDR scores were ≥1.0 were classified as ‘converters’ (*n* = 105); otherwise, they were classified as ‘nonconverters’ (*n* = 216).

Aβ42, total tau (t-Tau) and p-Tau levels in cerebrospinal fluid (CSF) were measured using the multiplex xMAP Luminex platform (Luminex, Austin, TX, USA) with INNOBIA AlzBio3 (Innogenetics, Ghent, Belgium) immunoassay kit-based reagents [24, 25]. Further details of ADNI methods for CSF acquisition and CSF measurement can be found at https://adni.loni.usc.edu/methods/. These data are available for 224 ADNI-1 participants (Fig. 1, Table S1).

### Selection of AD-related genes and SNPs

Among more than 30 gene loci for AD risk identified by GWAS [10–12, 15], 19 have been reported to be related to inflammation [11, 14, 15, 26–33]. Among these, we chose to study the SNPs of 10 genes based on the following criteria: (1) minor allele frequency (MAF) greater than 5%; (2) was the most significantly associated SNP with AD in the locus; and (3) showed evidence of replication in independent studies (Table S2).

### Genotyping, quality check and genotype imputation

Genotype calling and imputation in the UKBB dataset were performed as previously described [34]. SNP genotype data for the FHS cohort that were previously filtered and imputed were obtained from the Trans-Omics for Precision Medicine (TOPMed) Imputation Server (https://imputation.biodatacatalyst.nhlbi.nih.gov/#!). We imputed genotypes for ADNI participants using the TOPMed reference panel. The imputation quality *r*^2^ of all 10 SNPs was >0.95. The *APOE* genotype for UKBB subjects was determined by the combination of rs7412 and rs429358 alleles that define the ε2, ε3, and ε4 isoforms using a pipeline we developed previously (https://github.com/jjfarrell/apoe-genotyper). *APOE* genotypes for FHS and ADNI subjects were determined using TaqMan assays for these two SNPs. Details of the SNPs included in this study are shown in Table S2.

### Serum CRP measurement

CRP (high sensitivity, hs-CRP) was measured in the period 2006–2010 and a second time in 2012–2013 in UKBB subjects by immunoturbidimetric-high sensitivity analysis on a Beckman Coulter AU5800. Measurements obtained during the first period were used as the baseline level for this study. Details have been previously described (http://biobank.ctsu.ox.ac.uk/crystal/label.cgi?id=17518; http://biobank.ctsu.ox.ac.uk/crystal/field.cgi?id=30710; https://biobank.ctsu.ox.ac.uk/showcase/showcase/docs/biomarker_issues.pdf). CRP levels in FHS participants were determined using a Dade Behring BN100 nephelometer [35] from fasting blood samples that were collected at examination 7 from the antecubital vein when the participants were supine. Plasma samples were obtained from ADNI participants as previously described [36] and assessed using the Human DiscoveryMAP Panel and measurement platform including CRP protein and >100 other proteins as described [37]. Multiple CRP cutoffs (3–12 mg/L) were used to define low-grade inflammation [8].

### Statistical analysis

Analyses were performed using the R statistical environment (R 3.6.2) and python 3.7.7 hail module (https://github.com/hail-is/hail/commit/582b2e31b8bd). Several statistics, including the number of subjects, age, sex, years of education, *APOE* ε4 status, CRP level and AD status, were summarized as the basic characteristics of the stratified population under different CRP cutoffs (3, 8–12 mg/L). Group differences were assessed by analysis of variance (ANOVA) for normally distributed continuous variables, by the Kruskal–Wallis rank sum test for continuous variables with skewed distributions, and by the χ^2^ test for categorical variables.

Cox proportional hazards regression analysis was initially performed by including SNP minor allele dosage (0, 1, 2), CRP level, and a term for the interaction between SNP and CRP level, as well as several covariates, including age at CRP measurement (baseline), sex, years of education, and 6 principal components (PCs) of ancestry that were associated with AD status (*p* < 0.05). A model including an additional term for the presence or absence of the *APOE* ɛ4 allele was also tested and showed no meaningful differences. Specifically, the *APOE* ɛ4 group includes *APOE* ɛ3ɛ4 and *APOE* ɛ4ɛ4, while the non-*APOE* ɛ4 group includes *APOE* ɛ2ɛ2, *APOE* ɛ2ɛ3 and *APOE* ɛ3ɛ3. In addition, Kaplan‒Meier survival analysis and Cox proportional hazards regression models were applied to evaluate the association of SNPs and high CRP status defined at different cutoffs with incident AD. Nominally significant results (*p* < 0.05 in interaction tests and stratified genotypes analysis) were further pursued by applying the same Cox proportional hazards regression models to the FHS (incident AD) and ADNI data (MCI to AD conversion). Biomarkers such as Aβ and tau were also analyzed as supportive evidence in ADNI1 using linear regression models adjusted for age at baseline, sex, years of education and *APOE* ε4. Stratification analyses using different coding of genotypes and different CRP cutoffs were performed for significant SNPs. Effect estimates of AD incidence from the UKBB and FHS datasets were combined by inverse-variance weighted meta-analysis using METAL [38]. Power analysis was conducted for stratified genotype analysis, and the results are provided in Table S3.

## Results

### Characteristics of the study population

The 279,176 UKBB participants included in this study (Table 1) had an average follow-up period of 8.3 years, and 803 of them developed AD (mean age at onset = 70.5 years). Subjects with CRP concentrations ≥8 mg/L (*n* = 17,173, 6.2%) compared to those with a lower concentration of CRP were slightly older (*p* < 0.001) and more likely to be female (59.2% vs. 53.9%, *p* < 0.001) and *APOE* ε2 carriers (15.5% vs. 13.4%, *p* < 0.001) and were less likely to be *APOE* ε4 carriers (17.3% vs. 24.2%, *p* < 0.001). The incidence of AD was slightly higher in the group with CRP ≥ 8 mg/L than in the CRP < 8 mg/L group (0.4% vs. 0.3%, *p* = 0.10), but the difference was not significant. The age at onset of AD was similar between these CRP groups (*p* = 0.14, 95% CI = [−1.75, 0.26] for CRP 8 mg/L cutoff).

SNPs displaying nominal interaction with CRP for AD risk in the UKBB cohort were further evaluated in 2,853 cognitive normal control subjects and 156 AD incident cases in the FHS cohort (Table S1). Compared with UKBB subjects, FHS subjects had a similar age at baseline exam 7 (60.8 vs. 60.1) and an older AD onset age (80.9 vs. 70.5). In addition, FHS subjects had a higher average CRP level (2.2 mg/L vs. 1.4 mg/L), lower *APOE* ε4 carrier frequency (21.9% vs. 24.2%), and higher proportion of individuals with AD (5% vs. 0.3%) than UKBB subjects. In addition, the follow-up period from age at CRP measurement to censoring age was longer for FHS than UKBB subjects (average 14.93 vs. 8.45 years). However, the associations of CRP concentration with age, female sex, lower *APOE* ε4 carrier frequency and AD risk were similar to those observed in UKBB subjects.

### Impact of peripheral CRP on the association of established AD risk loci with AD incidence

**Fig. 2:**
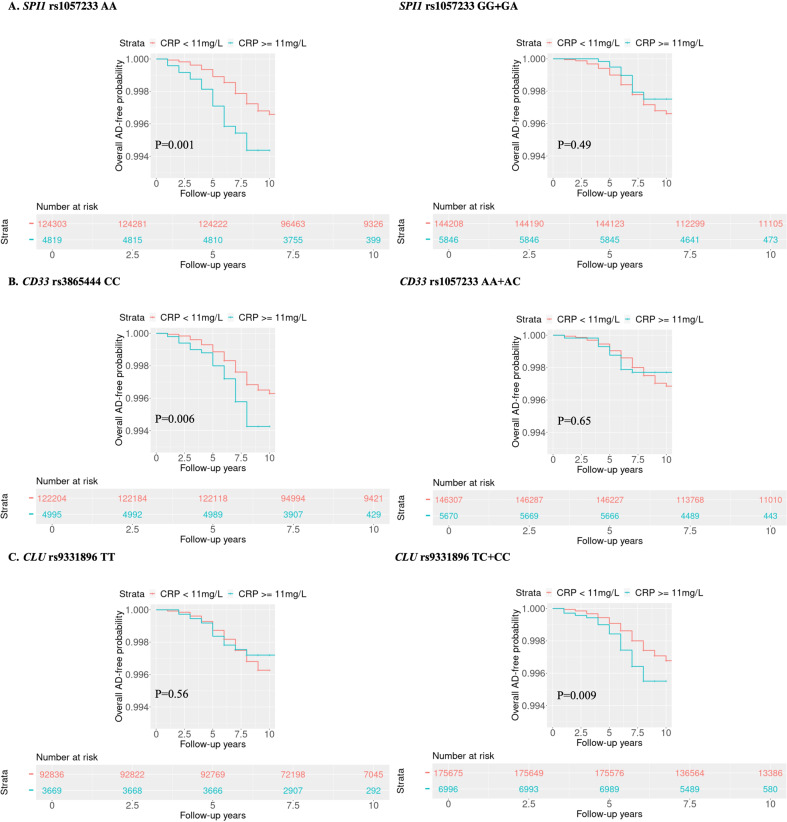
Kaplan‒Meier analysis in UKBB for AD-free probability under different CRP levels (mg/L) among genotypes in 3 SNPs. **A** *SPI1* rs1057233*-*AA vs. rs1057233-GG + GA. **B** *CD33* rs3865444-CC vs. rs3865444-AA + AC. **C** *CLU* rs9331896-TT vs. rs9331896-CC + CT genotypes. Red: CRP < 11 mg/L, Green: CRP ≥ 11 mg/L. Raw P values are presented.

**Fig. 3:**
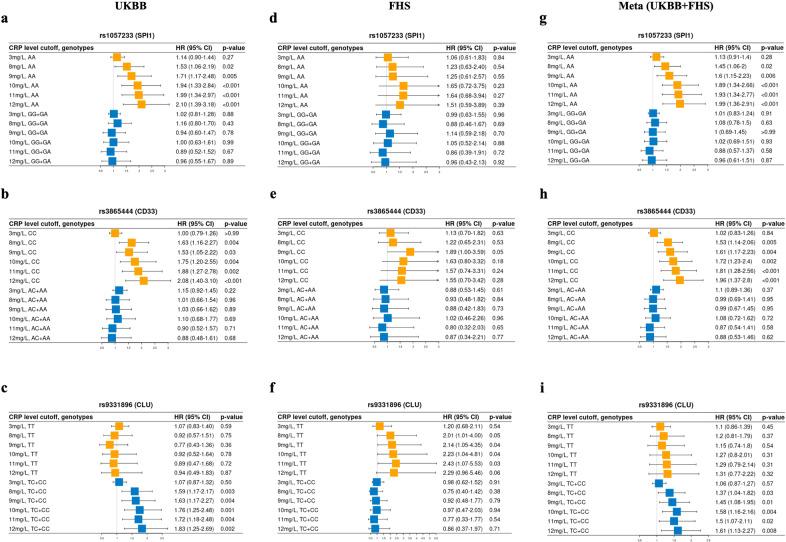
Forest plots of the results from UKBB, FHS and meta-analysis for the stratified genotype analysis of 3 SNPs for the effect of CRP levels on AD incidence. The Cox proportional hazard regression models were applied to estimate the effect of different levels of serum CRP on the incidence of AD among different genotypes of *SPI1, CD33*, and *CLU* after adjusting for age, sex, years of education, *APOE* ε4 and PCs. The results from UKBB are shown **(a**–**c)**, and those from FHS are shown **(d**–**f)**. The results from the meta-analyses of UKBB and FHS are shown (**g**–**i**). Raw *P* values are presented. For all CRP cutoffs from 3–12 mg/L, refer to Fig. S2.

SNPs at four of the 10 tested loci (*HLA-DRB1* rs9271192*, CLU* rs9331896, *ADAM10* rs593742 and *CD33* rs3865444) were significantly associated with AD risk in the UKBB dataset. However, only the *SPI1* SNP rs1057233 showed nominal evidence of an interaction with CRP concentration on AD risk (*p* = 0.03) (Table 2). The results were unchanged by removing the ɛ4 carrier status from the model (data not shown). Further examination of these five SNPs using different CRP cutoffs to define high chronic inflammation revealed evidence of nominally significant interactions (*p* < 0.05) between high CRP levels (9–12 mg/L) and SNPs in *CLU, SPI1* and *CD33* for AD risk (*p* < 0.05) (Table S4). Kaplan‒Meier analyses investigating the effect of the interaction of CRP concentration with these three SNPs highlighted significantly lower AD-free probability among subjects having CRP greater than 11 mg/L and at least one of the following genotypes: *SPI1* rs1057233-AA (*p* = 0.001), *CD33* rs3865444-CC (*p* = 0.006) or *CLU* rs9331896-CC/CT (*p* = 0.009) (Fig. 2). In contrast, AD risk was not influenced by elevated CRP among persons with other genotypes for these SNPs. Further stratification analysis showed that among subjects with rs1057233-AA and rs3865444-CC genotypes, the hazard ratio for AD was progressively larger with increasing CRP concentration (*p* < 0.001, Fig. 3, Fig. S1, Fig. S2, Table S5). All CRP cutoff (3–12 mg/L) results are presented in Fig. S2. A similar but marginally significant trend was noted for rs9331896-CC/CT genotypes among UKBB but not FHS subjects. The significance of the interactions involving the *SPI1* and *CD33* SNPs increased slightly in the meta-analysis of UKBB and FHS results compared to those for UKBB subjects alone (Fig. 3g, h).

**Table 2:** Interactions between 10 SNPs (MAF > 5%) and continuous CRP levels on AD risk using the Cox proportional hazards regression model in UKBB.

Gene Locus	Chr:Pos (GRCh37)	Major Allele	Minor Allele	dbSNP ID	Function	MAF	Discovery UK-biobank			
							Main Effect of SNP^a^		Interaction Effect (SNP:CRP)^b^	
							HR (95%CI)	P value^c^	HR (95%CI)	P value^c^
*CR1*	1:207692049	G	A	rs6656401	Intron	0.18	1.12 (0.99–1.27)	0.06	1.01 (0.99–1.04)	0.43
***HLA-DRB1***	**6:32578530**	**A**	**C**	**rs9271192**	**Intergenic**	**0.27**	**1.19 (1.07–1.32)**	**0.002**	1.02 (0.99–1.04)	0.18
*CD2AP*	6:47487762	A	G	rs10948363	Intron	0.27	1.02 (0.91–1.14)	0.73	1.00 (0.98–1.03)	0.78
*EPHA1*	7:143109139	T	C	rs11767557	Intron	0.21	1.02 (0.91–1.15)	0.72	0.99 (0.96–1.02)	0.58
***CLU***	**8:27467686**	**T**	**C**	**rs9331896**	**Intron**	**0.41**	**0.90 (0.81–0.99)**	**0.03**	1.01 (0.98–1.03)	0.67
***SPI1***	**11:47376448**	**A**	**G**	**rs1057233**	**3’ UTR**	**0.32**	1.00 (0.90–1.12)	0.93	**0.97 (0.95–0.99)**	**0.03**
*MS4A6A*	11:59923508	A	G	rs983392	Intergenic	0.41	0.93 (0.84–1.03)	0.14	0.99 (0.97–1.02)	0.64
***ADAM10***	**15:59045774**	**A**	**G**	**rs593742**	**Intron**	**0.31**	**0.89 (0.8–0.99)**	**0.04**	1.00 (0.98–1.03)	0.94
*ABCA7*	19:1063443	G	A	rs4147929	Intron	0.18	1.06 (0.93–1.2)	0.38	1.00 (0.98–1.03)	0.83
***CD33***	**19:51727962**	**C**	**A**	**rs3865444**	**5’UTR**	**0.32**	**0.83 (0.75–0.93)**	**<0.001**	0.98 (0.95–1.01)	0.16

Using the UK Biobank (UKBB) dataset and Cox proportional hazards regression, 10 SNPs related to AD and inflammation were chosen, and their relationships and interactive effects with continuous CRP levels for AD risk were examined. Raw *P* values are shown.

*AD* Alzheimer’s disease, *CRP* C-reactive protein, *HR* hazard ratio, *Chr* chromosome, *Pos* position, *MAF* minor allele frequency.

^a^Logistic regression for the relationship between SNP dosage alone and AD without including CRP.

^b^Cox proportional hazards regression models for the interaction: SNP dosage ∗ CRP level after adjusting for age, sex, years of education, *APOE* ε4 status and PCs. Raw *P* values are shown.

^c^Raw *P* values.

Bold value indicates *P* < 0.05.

### Association of the interaction between SPI1 and CD33 genotypes and CRP levels with MCI to AD conversion and CSF AD biomarkers

Next, we investigated whether the interactions of CRP levels with *SPI1* and *CD33* polymorphisms, which were associated with AD risk in the UKBB and FHS datasets, were also associated with the conversion of MCI to AD and AD-related CSF biomarkers in ADNI participants. At high CRP levels, the trends of association of the interaction between CRP and the *SPI1* (*p* = 0.03) and *CD33* (*p* = 0.07) SNPs with MCI-to-AD conversion were in the same direction as observed for the association of these SNPs with AD risk in the UKBB and FHS datasets (Table S6).

**Fig. 4:**
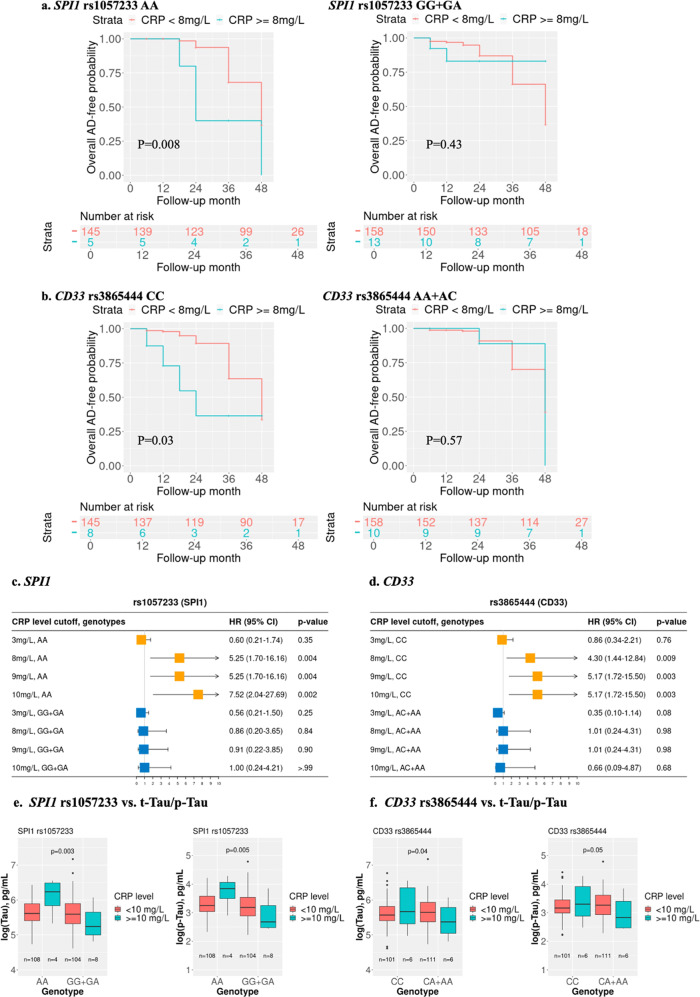
Kaplan‒Meier survival plots and forest plots using the ADNI cohort for the stratified genotype analysis for the effect of CRP levels on MCI-AD conversion in the Cox proportional hazard regression models and boxplots for CRP-SNP interaction effects on CSF biomarkers (t-Tau and p-Tau): *SPI1* rs1057233 and *CD33* rs3865444. ADNI MCI participants were stratified by genotypes. Kaplan‒Meier survival plots were generated for AD free time for *SPI1* rs1057233 and *CD33* rs3865444 genotypes; Red: CRP < 8 mg/L, Green: CRP ≥ 8 mg/L (**a**, **b**). Forest plots with the estimated effect of different levels of serum CRP on the MCI-to-AD conversion among different genotypes after adjusting for age, sex, education and *APOE* ε4 (**c**, **d**). ADNI participants with measured CSF AD biomarkers were stratified by genotype. t-Tau and p-Tau measured at the last exam were log transformed and are shown in boxplots. p values of the interaction between CRP and *SPI1* rs1057233/*CD33* rs3865444 genotypes were calculated using linear regression analysis after adjusting for age, sex, education and *APOE* ε4. Red: CRP < 10 mg/L, Green: CRP ≥ 10 mg/L (**e**, **f**). Raw *P* values are presented.

Survival analysis conducted separately among subjects with CRP levels less than 8 mg/L and 8 mg/L or greater revealed that conversion from MCI to AD was impacted only among those with the *SPI1* rs1057233-AA or *CD33* rs3865444-CC genotypes (Fig. 4a, b). Similar findings were obtained from Cox proportional hazards analyses that adjusted for age, sex, years of education, and *APOE* ɛ4 (*p* < 0.01) (Fig. 4c, d).

Amyloid plaque represented by amyloid beta (Aβ) levels and neurofibrillary tangles represented by tau levels are two neuropathological hallmarks of AD. PET imaging or cerebral spinal fluid (CSF)-measured Aβ and tau is considered the gold standard for the in vivo diagnosis of AD, as recently proposed in the amyloid-tau-neurodegeneration (A/T/N) framework [39]. Further analyses of individuals with these genotypes showed that rs1057233-AA subjects with higher levels of CRP, particularly above the 10 mg/L cutoff, had higher levels of t-Tau (interaction *p* = 0.003) and p-Tau (interaction *p* = 0.005), but these effects were not observed in those with other rs1057233 genotypes regardless of CRP concentration (Fig. 4e, Table 3). Similar patterns were observed for rs3865444-CC subjects with higher CRP levels, but the results were much more attenuated (Fig. 4f, Table 3, Fig. S3). The interactions of CRP level with the *SPI1* and *CD33* SNPs were not associated with Aβ_42_ level (Table 3).

**Table 3:** Interaction between SNPs and high CRP on cerebral spinal fluid ABeta42, t-Tau, p-Tau in the ADNI study.

CRP level^a^	Interaction Effect for CSF Biomarkers	rs1057233 (*SPI1*)^a^		rs3865444 (*CD33*)^a^	
		Estimate (SE)	P value^a^	Estimate (SE)	P value^a^
3 mg/L cutoff	Abeta	−0.153 (0.181)	0.40	−0.316 (0.172)	0.07
	t-Tau	−0.146 (0.136)	0.28	−0.069 (0.130)	0.55
	p-Tau	−0.167 (0.154)	0.27	−0.046 (0.147)	0.70
8 mg/L cutoff	Abeta	0.211 (0.281)	0.46	−0.463 (0.265)	0.08
	t-Tau	−0.474 (0.213)	**0.03**	−0.351 (0.201)	0.07
	p-Tau	−0.520 (0.241)	**0.04**	−0.369 (0.227)	0.09
9 mg/L cutoff	Abeta	0.122 (0.290)	0.68	−0.465 (0.279)	0.10
	t-Tau	−0.481 (0.220)	**0.03**	−0.420 (0.213)	**0.04**
	p-Tau	−0.512 (0.249)	**0.04**	−0.434 (0.242)	0.06
10 mg/L cutoff	Abeta	‘0.038 (0.319)	0.90	−0.324 (0.301)	0.28
	**t-Tau**	−0.717 (0.238)	**0.003**	−0.451 (0.227)	**0.04**
	**p-Tau**	−0.766 (0.270)	**0.005**	−0.492 (0.257)	0.05

Different CRP cutoffs ≥3, 8, 9 and 10 mg/L were used. General linear regression (GLM) was used to study the relationships between these blood CRP cutoffs and two SNPs for AD biomarkers, including Abeta42, total Tau (t-Tau) and phosphorylated Tau (p-Tau), in cerebrospinal fluid (CSF). The models were adjusted for age, sex, years of education and APOE ɛ4. Prevalent AD was removed. Biomarkers at each last exam were used. Raw *P* values are shown.

^a^Dummy variable for SNPs and CRP in Model: rs1057233: GG + GA = 1, AA = 0; rs3865444: CA + AA = 1, CC = 0; CRP cutoff code: < cutoff=0; ≥ cutoff=1.

^b^Raw *P* values.

Bold value indicates *P* < 0.05.

## Discussion

The results of this study extend our previous finding that elevated CRP impacts AD risk among *APOE* ε4 carriers [8] and the well-established relationship between inflammation and AD [40–42]. We found evidence in three independent datasets that the association of AD with SNPs in the neuroinflammatory AD-associated genes *SPI1* and *CD33* is modulated by CRP levels. Elevated CRP levels have a larger effect size for MCI-to-AD conversion than AD incidence in cognitively normal individuals among the affected genotypes. Consistent with the effect of the interaction between *APOE* ε4 and CRP on CSF AD biomarkers [43], this study also demonstrated that the combination of elevated CRP and AD-associated genotype in *SPI1* or *CD33* was associated with increased CSF t-Tau and p-Tau levels. Taken together, our findings suggest that CRP produced during peripheral chronic inflammation may play a key role in modulating the effects of *APOE*, *SPI1* and *CD33* genotypes on AD risk.

One possible explanation for the association of the interactions of CRP with these genes with AD is that the SNPs contribute to AD risk through their influence on CRP levels. However, *SPI1*, *CD33* and *CLU* were not found to be significantly associated with CRP levels in a GWAS including more than 200,000 individuals [44]. Rather, the findings from our analyses, which considered multiple CRP cutoffs used to evaluate inflammation severity in clinical practice, may provide insight into mechanisms linking *SPI1* and *CD33* to AD. It is possible that the effect of peripheral chronic low-grade inflammation on AD is influenced by *SPI1* and *CD33* genotypes. Elderly persons more frequently suffer from bacterial and viral infection/inflammation as well as obesity and cardiovascular diseases in peripheral systems, which lead to elevated CRP levels. The relationship between high blood CRP and AD risk is controversial [45–47], probably due to unaccounted for interactions of CRP with AD-related genes involved in inflammation, inflammatory stage, and treatment for inflammation. It is possible that severe and chronic peripheral inflammation caused by persistent bacterial or viral infection may enhance certain genetic vulnerabilities for AD, including those conferred by *APOE* ε4, as well as particular *SPI1* and *CD33* genotypes. Given that our recent study identified monomeric CRP as a mediating factor for *APOE* ε4-related AD pathogenesis [48], this study suggests that CRP may also be such a mediating factor in the AD pathophysiological process related to *SPI1* or *CD33*.

*SPI1* encodes the ETS-domain transcription factor PU.1, which is critical for myeloid cell development and is a major regulator of microglial gene expression. AD heritability (measured by summary statistics from IGAP GWAS [30]) was enriched within the PU.1 cistrome, implicating a myeloid PU.1 target gene network in AD [14]. The *SPI1* SNP rs1057233 was reported to affect its expression and influence chronic autoimmune disease [49]. In the brain, PU.1 is specifically expressed in microglia, and recent evidence suggests that reductions in PU.1 contribute to a delayed onset of AD, possibly by limiting neuroinflammatory responses [50]. Therefore, we reasoned that *SPI1* risk allele carriers have higher expression of PU.1, leading to enhanced neuroinflammatory responses to peripheral chronic inflammation, which may increase their risk of AD. Alternatively, high CRP levels may disproportionately increase *SPI1* expression among *SPI1* risk allele carriers, resulting in PU.1 accumulation and subsequently increasing AD risk.

As one of the key microglial receptors, *CD33* is involved in the innate immune pathway associated with anti-inflammatory signaling and hematopoietic cell lineage [17]. *CD33* also plays a role in mediating cell‒cell interactions and maintaining immune cells in a resting state [51–53]. *CD33* is an innate immune effector of neuroinflammation. *CD33* controls the microglial activation state, turning microglia from housekeepers that clear amyloid into killers that destroy neurons [54–57]. Its association with AD is supported by some [10, 16, 58–61], but not all [9, 28] genetic studies. The *CD33* rs3865444(C) risk allele was reported to be associated with greater cell surface expression of *CD33* in monocytes, accumulation of amyloid pathology and increased numbers of activated human microglia [62], and increased *CD33* expression was also observed in microglial cells in the AD brain [56]. Consistent with results from a study showing that deletion of *CD33* results in an inflammatory human microglial phenotype [63], our results suggest that a high CRP level together with the rs3865444(C) risk allele increases *CD33* expression in the brain and, by extension, neuroinflammation, leading to hallmark AD pathology. Most recently, Griciuc et al. reported that gene therapy for AD targeting *CD33* reduces amyloid beta accumulation and neuroinflammation [64]. Alector has developed the mAb AL003, which blocks *CD33* function and may reduce neuroinflammation in the AD brain and is in early-phase clinical trials for AD [54]. Our study suggests that stratification of patients based on their peripheral chronic inflammation severity (i.e., CRP level) and genotypes can be very helpful to improve personalized AD intervention and treatment.

Age is known as a risk factor for both AD development and peripheral chronic inflammation. Neuroinflammation clearly occurs in pathologically vulnerable regions of the AD brain. Infections of the respiratory, gastrointestinal, and urinary tract systems as well as cardiovascular diseases and diabetes are common in older adults and can trigger chronic low-grade inflammation (i.e., high CRP levels). Such an inflammatory response may increase susceptibility to AD, especially among those carrying genetic risk variants. As CRP activates the complement system [65] and the activated complement system is involved in AD pathogenesis [66], all three genes, SPI1 [67], CD33 [68] and CLU [69], are linked with complement in proinflammation, suggesting a common cross-shared pathway for all of these factors in AD. Since it is currently unrealistic to change genetic risk polymorphisms, modifying or treating mediating factors such as CRP by pharmacological and nonpharmacological approaches for genetic risk carriers of *APOE* ε4, *SPI1* rs1057233(AA) and *CD33* rs3865444(CC) could be an alternative strategy to prevent or treat AD [70].

This study has several limitations. *CLU* (clusterin) is also known as apolipoprotein J. It is reported that the *CLU* polymorphism influences its expression, which is increased in inflammatory states [71]. We found that elevated CRP impacted the *CLU* rs9331896(C) allele on AD risk in UKBB only. In addition, the imbalanced size for the ADNI cohort within high CRP cutoffs may have skewed the findings. Additional large independent AD human cohorts are likely needed to further characterize this relationship. Second, although we obtained significant results after a stringent Bonferroni multiple-testing correction (i.e., *p* < 0.0028), which is 0.05/18 independent tests (i.e., 3 SNPs*6 CRP cutoffs) (Fig. 3), we still might not catch those with chronic and longitudinal inflammation with one CRP measurement without having longitudinal measurements of CRP. Finally, UKBB had a relatively lower AD incidence rate than many other cohorts reported. We reasoned that in UKBB, the population evaluated had a shorter follow-up time and that more AD incident cases would be observed when the study continued to evolve [72]. Nevertheless, our study using three different cohorts suggests a role for the clinical biomarker CRP used under infection or inflammation conditions in monitoring risk in those with specific genetic factors for AD development as well as a role in precision medicine-based drug development. Future studies in larger cohorts with frequent longitudinal monitoring of serum CRP are needed to validate our findings, and the use of multiethnic cohorts will be necessary to test the generalizability of our findings.

## Supplementary information

Supplemental Figures

Supplementary Tables
