# Supplementary material for: The impact of increasing levels of blood C-reactive protein on the inflammatory loci SPI1 and CD33 in Alzheimer’s disease
Source: Transl Psychiatry. 2022 Dec 22;12:523. doi: 10.1038/s41398-022-02281-6 (PMC9780312; doi:10.1038/s41398-022-02281-6)
Supplement: Supplementary file 1 — Supplemental Figures [file 41398_2022_2281_MOESM1_ESM.docx]

**Supplement Materials**

**Supplement tables (1-6) are in supplement_table.xlsx**

**Supplementary Table 1.** Basic characteristics in FHS and ADNI

**Supplementary Table 2.** Information for 10 inflammatory & AD-related SNPs^1-11^

**Supplementary Table 3.** Power analysis for the stratified genotype analysis

**Supplementary Table 4.** Interaction effect between high CRP cutoff and SNPs genotypes (dominant) on AD risk, adjusted for age, sex, years of education, APOE4 and PCs, for 5 SNPs in UKBB, FHS and meta-analysis

**Supplementary Table 5.** Number of AD cases (percentage) under different CRP level cutoff for 3 significant SNPs in UKBB

**Supplementary Table 6.** Interaction of CRP and SPI1 rs1057233/CD33 rs3865444 on MCI-AD conversion, adjusted by age, sex, years of education and APOE4 in ADNI MCI patients

**Supplement Figure 1.** Trends of cumulative AD incidence rates across CRP level cutoff (1-12 mg/L) among 3 SNPs genotypes in UKBB.

**Supplement Figure 2.** Forest plot for the stratified genotype analysis of 3 SNPs for the effect of CRP levels cutoffs (all 3-12 mg/L) on AD incidence in Cox proportional hazard regression models.

**Supplement Figure 3.** CSF AD biomarkers based on elevated serum CRP and different genotypes of *SPI1* and *CD33*.

**Supplement Figure 1.** Trends of cumulative AD incidence rates across CRP level cutoff (1-12 mg/L) among 3 SNPs genotypes in UKBB.

1. **CLU**

**
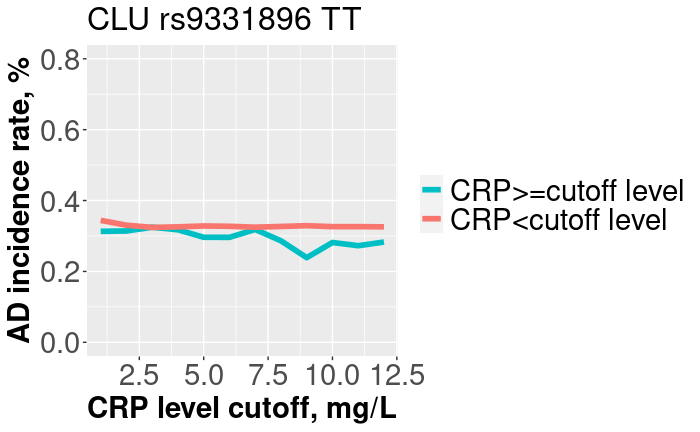

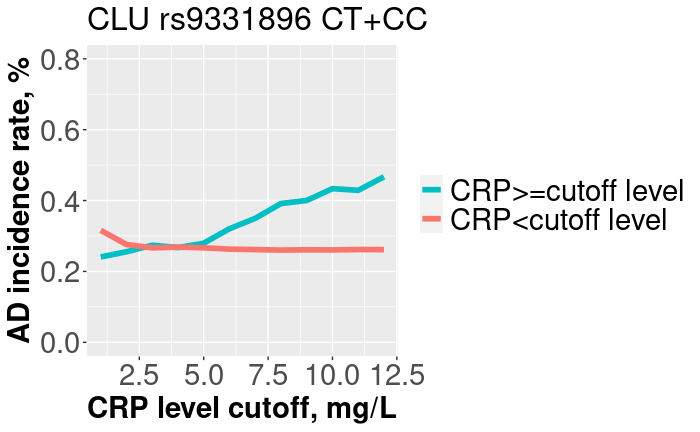
**

1. **SPI1**

**
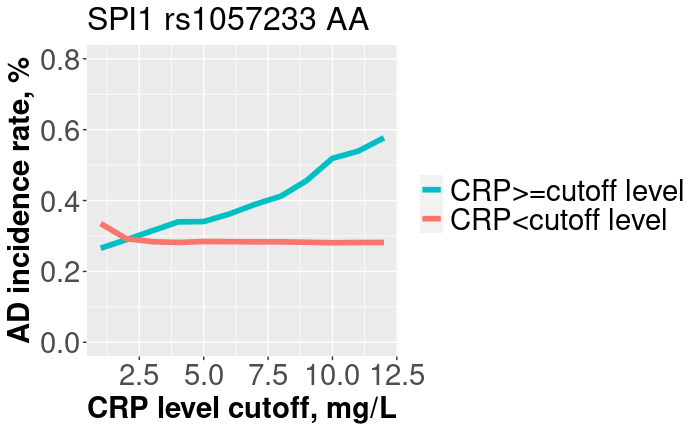

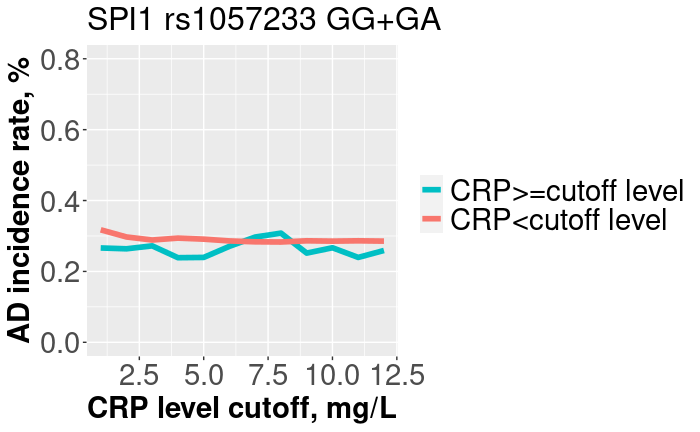
**

1. **CD33**

**
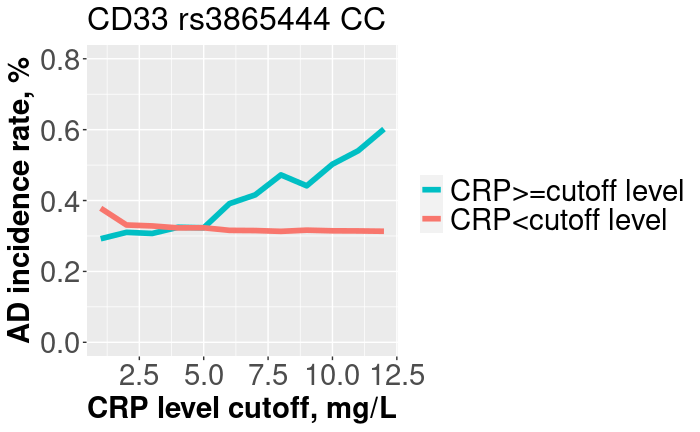
**
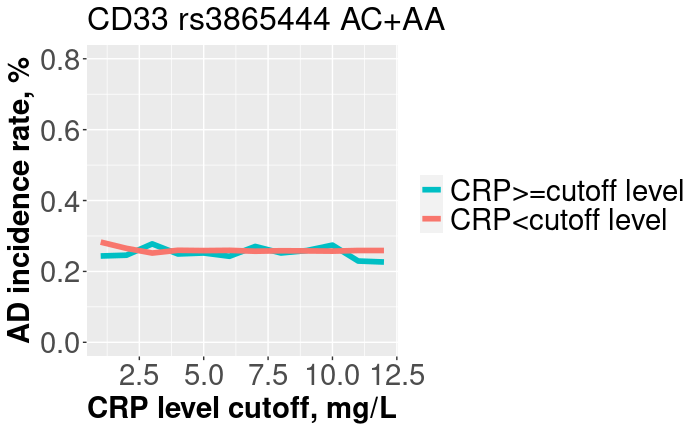


**Supplement Figure 2.** Forest plot for the stratified genotype analysis of 3 SNPs for the effect of CRP levels cutoffs (all 3-12 mg/L) on AD incidence in Cox proportional hazard regression models.

**Supplement Figure 3.** CSF AD biomarkers based on elevated serum CRP and different genotypes of *SPI1* and *CD33*. ADNI participants with measured CSF AD biomarkers were divided into *SPI1* AA/GG+GA and *CD33* CC/AA+AC genotypes. Different CRP cutoff level (3-10mg/L) were used to further divide participants into two genotype groups. Prevalent AD cases were excluded. t-Tau and p-Tau at each participants’ last exam were used. Level of t-Tau and p-Tau were log transformed. Boxplots of t-Tau and p-Tau for different SNP-CRP groups with p-values of interaction between CRP cutoffs and SNPs genotypes from linear regression analysis, adjusted for age, sex, education and *APOE* ε4, were shown for *SPI1* (**a-b**) and *CD33* (**c-d**).

1. **SPI1 vs. t-Tau**

1. **SPI1 vs. p-Tau**


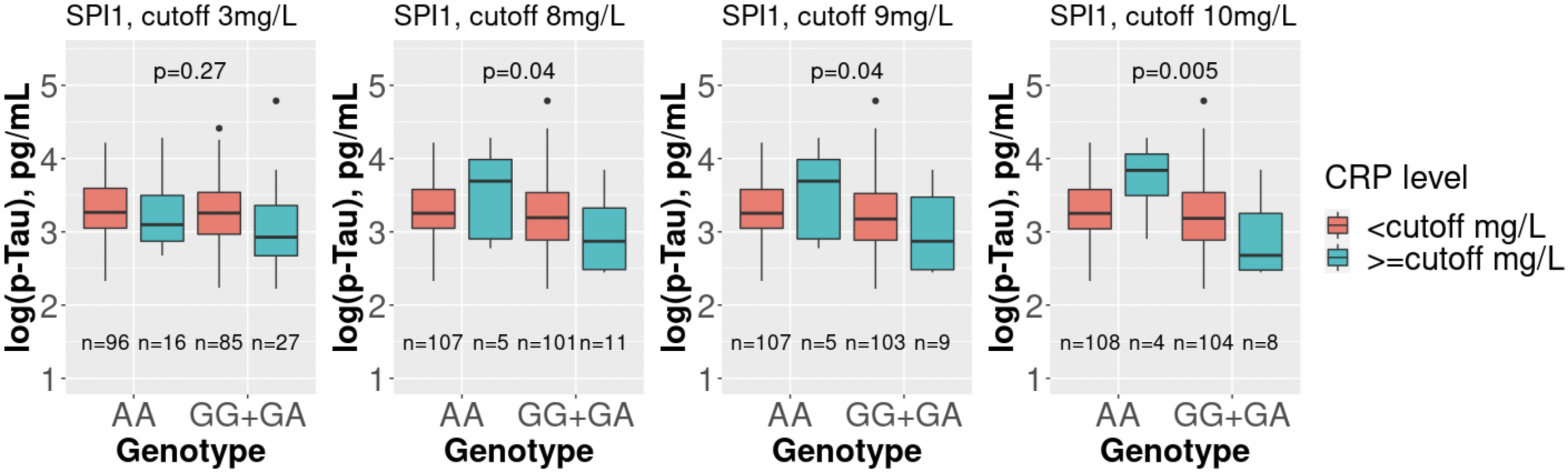


1. **CD33 vs. t-Tau**

**CRP**


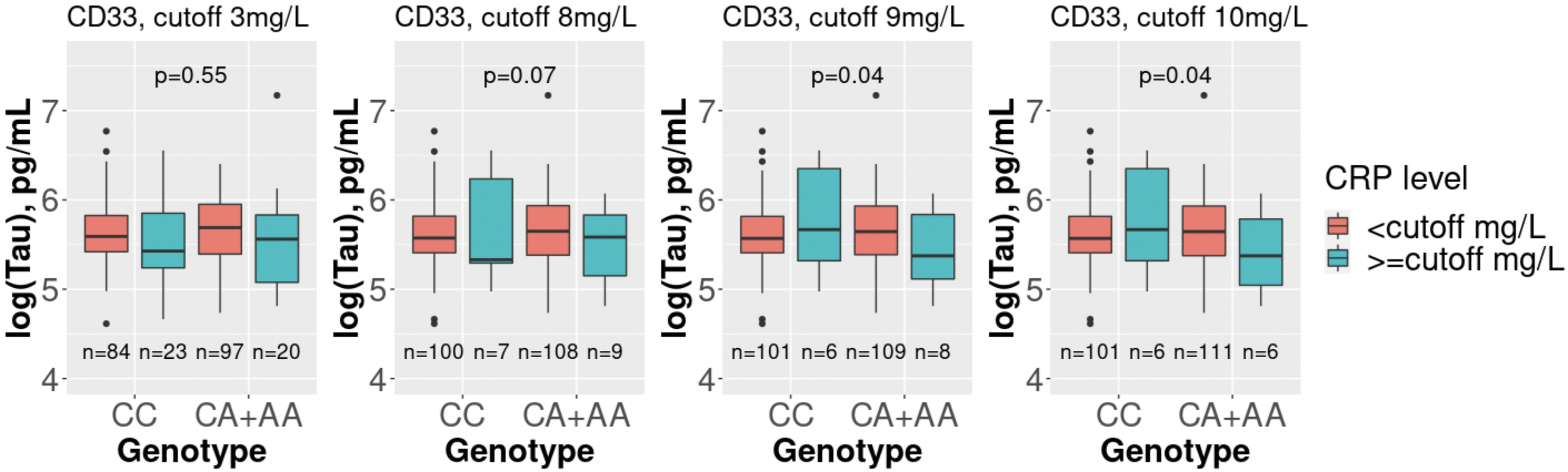


1. **CD33 vs. p-Tau**


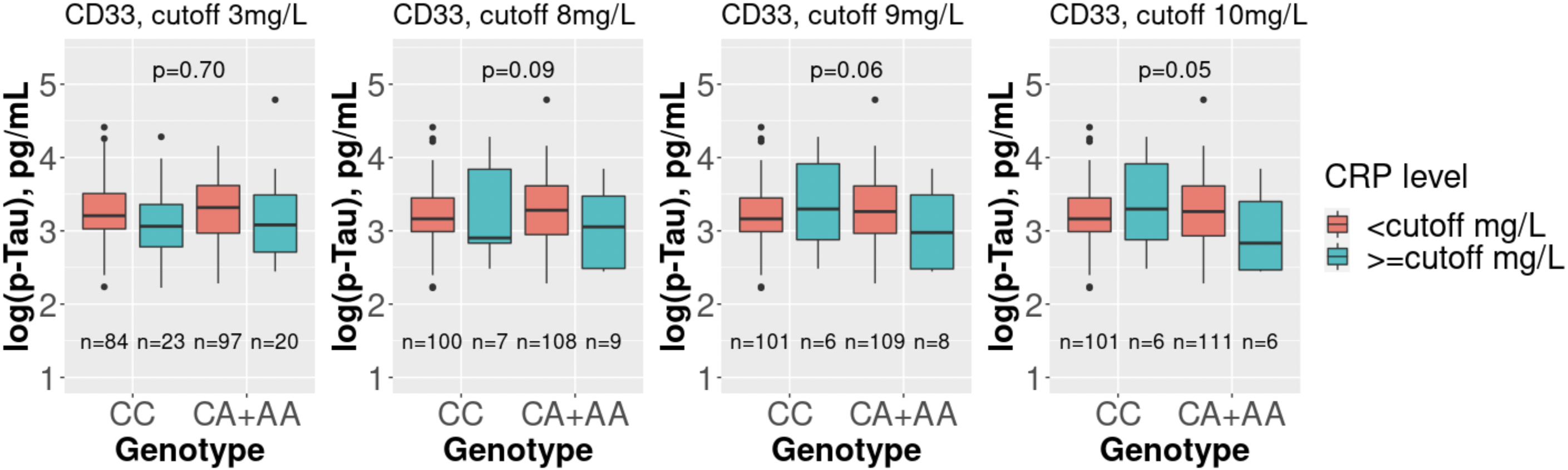


**Reference**

1. Hollingworth P, Harold D, Sims R, et al. Common variants at ABCA7, MS4A6A/MS4A4E, EPHA1, CD33 and CD2AP are associated with Alzheimer's disease. *Nat Genet*. May 2011;43(5):429-35. doi:10.1038/ng.803

2. Lambert JC, Ibrahim-Verbaas CA, Harold D, et al. Meta-analysis of 74,046 individuals identifies 11 new susceptibility loci for Alzheimer's disease. *Nat Genet*. Dec 2013;45(12):1452-8. doi:10.1038/ng.2802

3. Malik M, Parikh I, Vasquez JB, et al. Genetics ignite focus on microglial inflammation in Alzheimer's disease. *Mol Neurodegener*. Oct 5 2015;10:52. doi:10.1186/s13024-015-0048-1

4. Villegas-Llerena C, Phillips A, Garcia-Reitboeck P, Hardy J, Pocock JM. Microglial genes regulating neuroinflammation in the progression of Alzheimer's disease. *Curr Opin Neurobiol*. Feb 2016;36:74-81. doi:10.1016/j.conb.2015.10.004

5. Naj AC, Jun G, Beecham GW, et al. Common variants at MS4A4/MS4A6E, CD2AP, CD33 and EPHA1 are associated with late-onset Alzheimer's disease. *Nat Genet*. May 2011;43(5):436-41. doi:10.1038/ng.801

6. Kunkle BW, Grenier-Boley B, Sims R, et al. Genetic meta-analysis of diagnosed Alzheimer's disease identifies new risk loci and implicates Abeta, tau, immunity and lipid processing. *Nat Genet*. Mar 2019;51(3):414-430. doi:10.1038/s41588-019-0358-2

7. Dries JL, Kent SD, Virag JA. Intramyocardial administration of chimeric ephrinA1-Fc promotes tissue salvage following myocardial infarction in mice. *J Physiol*. Apr 1 2011;589(Pt 7):1725-40. doi:10.1113/jphysiol.2010.202366

8. Kim M, Suh J, Romano D, et al. Potential late-onset Alzheimer's disease-associated mutations in the ADAM10 gene attenuate {alpha}-secretase activity. *Hum Mol Genet*. Oct 15 2009;18(20):3987-96. doi:10.1093/hmg/ddp323

9. Gerhardt S, Hassall G, Hawtin P, et al. Crystal structures of human ADAMTS-1 reveal a conserved catalytic domain and a disintegrin-like domain with a fold homologous to cysteine-rich domains. *J Mol Biol*. Nov 2 2007;373(4):891-902. doi:10.1016/j.jmb.2007.07.047

10. Huang KL, Marcora E, Pimenova AA, et al. A common haplotype lowers PU.1 expression in myeloid cells and delays onset of Alzheimer's disease. *Nat Neurosci*. Aug 2017;20(8):1052-1061. doi:10.1038/nn.4587

11. Fagan V, Johansson C, Gileadi C, et al. A Chemical Probe for Tudor Domain Protein Spindlin1 to Investigate Chromatin Function. *J Med Chem*. Oct 24 2019;62(20):9008-9025. doi:10.1021/acs.jmedchem.9b00562
